# Alpha2B-Adrenergic Receptor Regulates Neutrophil Recruitment in MSU-Induced Peritoneal Inflammation

**DOI:** 10.3389/fimmu.2019.00501

**Published:** 2019-03-19

**Authors:** Lihua Duan, Jie Chen, Michael Razavi, Yingying Wei, Ying Tao, Xiaoquan Rao, Jixin Zhong

**Affiliations:** ^1^Department of Rheumatology and Clinical Immunology, Jiangxi Provincial People's Hospital, Nanchang, China; ^2^Cardiovascular Research Institute, School of Medicine, Case Western Reserve University, Cleveland, OH, United States; ^3^School of Medicine, Xiamen University, Xiamen, China; ^4^Oregon Institute of Occupational Health Sciences, Oregon Health & Science University, Portland, OR, United States

**Keywords:** gout, adrenergic receptor, neutrophil, monosodium urate, inflammation

## Abstract

Gout is one of the most common metabolic disorders in human. Previous studies have shown that the disease activity is closely associated with sympathetic nervous system (SNS). α_2B_-adrenergic receptor (α_2B_AR), a subtype of α2 adrenergic receptor, plays a critical role in many diseases. However, the role of α_2B_AR in the pathogenesis of gout remains unclear. Here, we assessed the role of α_2B_AR in the monosodium urate (MSU) crystals-induced peritonitis that mimics human gout by using the α_2B_AR-overexpressing mice (α_2B_AR-Tg). We found that the number of recruited neutrophils was significantly increased in the α_2B_AR-Tg mice after MSU treatment, when compared with wild type mice. In contrast, the number of macrophages was not changed. Importantly, there is no difference in the IL-1β levels and caspase-1 activity between wild type and α_2B_AR-Tg mice in the gout animal model. Notably, the enhanced neutrophil migration in α_2B_AR-Tg mice was dependent on the α_2B_AR overexpression in neutrophils, but not resulted from other tissues or cells with α_2B_AR overexpression. In conclusion, our data provide a direct evidence that α_2B_AR plays a critical role in neutrophil migration and MSU-induced inflammation.

## Introduction

The cross-talk between the sympathetic nervous system (SNS) and immune system has gained increasing attentions through catecholamines hormone (1–3). Catecholamines exert their physiological effects by binding adrenergic receptors, which are a class of G protein-coupled receptors. There are two main groups of adrenoreceptors, α and β, with 9 subtypes in total (4). An increased production of endogenous catecholamines epinephrine and norepinephrine can be observed in a status of anxiety, pathogenic challenge, and pain (5). It has been demonstrated recently that SNS play an important role in the induction of stress-induced pro-inflammatory cytokines, such as IL-1β induction (6). Consistent with this, the baseline epinephrine in healthy humans is inversely correlated with pro-inflammatory cytokine levels and positively with anti-inflammatory cytokine productions (7). In addition, SNS have also been implicated in chronic intestinal inflammation, multiple sclerosis, and rheumatoid arthritis through T cell immune responses (8–11).

Accumulating data indicate that SNS plays a critical role in the control of metabolic and cardiovascular homeostasis. Both experimental and clinical studies have shown that the changes of catecholamines in plasma and tissue were associated with the pathogenesis of metabolic and cardiovascular diseases (12, 13). Gout is one of the most common human metabolic diseases characterized by sudden pain and swelling in the joints. The abnormal uric acid (UA) metabolism results in monosodium urate (MSU) crystal formation in the joint and periarticular tissues, which activates the resident macrophage to produce IL-1β, a crucial inflammatory cytokines in the pathogenesis of gout (14, 15). Increasing evidences have demonstrated that α_2_AR stimulation promotes cytokine production. It has been shown that the α_2_AR was required for norepinephrine-induced DC endocytosis through modulating the PI3K and ERK1/2 signaling pathway in DCs (16). The α_2_AR agonist dexmedetomidine can decrease the IL-1β-induced IL-6 production in glioma cells (17). α_2_ARs include three highly homologous subtypes, α_2A_AR, α_2B_AR, and α_2C_AR. However, the modulation of α_2B_AR on the pathogenesis of gout remains elusive.

Here, we addressed the role of α_2B_AR in the pathogenesis of MSU-induced inflammation by using the α_2B_AR over-expressing transgenic mice (α_2B_AR-Tg). We found that the MSU-induced inflammation was exacerbated in α_2B_AR-Tg mice, characterized by increased neutrophils infiltration. In consistency, neutrophils with α_2B_AR over-expression also displayed an enhanced migratory ability in *in vitro* experiments. In contrast, the number of macrophage and IL-1β levels were not altered. In conclusion, α_2B_AR plays an important role in MSU-induced inflammation by enhancing the migration of neutrophils. Therefore, α_2B_AR may serve as a promising therapeutic target for gout.

## Materials and Methods

### Animals

FVB/NJ mice (wild-type, Wt) and α_2B_AR transgenic mice (α_2B_AR-Tg) in FVB/NJ background were purchased from the Jackson Laboratory. The mice were housed in the specific pathogen-free animal facility at the Case Western Reserve University. All experimental procedures involving mice were approved by the Institutional Animal Care and Use Committees (IACUCs) at the Case Western Reserve University and Xiamen University.

### Establishment of Acute Gout Animal Model

Male mice (6–8 weeks old) were administered an intraperitoneal (IP) injection of 3 mg MSU (Invivogen, US). After 16 h, the peritoneal exudate cells (PECs) and lavage fluids were harvested and subjected to flow cytometry analysis and cytokine assay.

### Flow Cytometry Analysis

The peritoneal exudate cells (PECs) were incubated with the fluorescent-conjugated monoclonal antibodies in the staining buffer. Antibodies used for flow cytometry are as follows, FITC anti-mouse F4/80, PE anti-mouse CD11b and PE/Cy7 anti-mouse Ly6G. All antibodies are purchased from Biolegend (San Diego, CA, US). Caspase 1 activity were determined by FAM-FLICA Caspase Assay Kits (Immunochemistry Technologies, Bloomington, MN, USA) according to the manufacturer's instructions through flow cytometry analysis.

### Mouse Bone Marrow-Derived Macrophage Generation

Bone marrow-derived macrophages (BMMs) were generated from the bone marrow cells as described previously (18). Briefly, bone marrow cells isolated from the animals were seeded at the concentration of 1 × 10^6^/ml cells and cultured in the presence of M-CSF (20 ng/ml) (Peprotech, US) for 7 days. The supernatant was replaced every 2 days. Adherent BMMs were harvested at day 7 for further study.

### Quantitative Real Time RT-PCR

Total RNA was extracted from PECs by TRIzol® Reagent (Invitrogen, Carlsbad, CA) as instructed. Reverse transcription of total RNA was performed using cDNA synthesis kit (ABI, ON, Canada). The primers for β-actin, IL-1β, MIP-2, NLRP3, and α_2B_AR gene are listed below. mip-2, forward: TCA ATG CCT GAA GAC CCT G, reverse: CCT TGA GAG TGG CTA TGA CTT C; adra2b, forward: CTG GGC TAT TGG TAC TTC TGG, reverse: AGT TGT ACT CCA ATG CTC GG; β-actin, forward: ACC TTC TAC AAT GAG CTG CG, reverse: CTG GAT GGC TAC GTA CAT GG; IL-1β, forward, ACG GAC CCC AAA AGA TGA AG, reverse: TTC TCC ACA GCC ACA ATG AG; nlrp3, forward: CTC CAA CCA TTC TCT GAC CAG, reverse: ACA GAT TGA AGT AAG GCC GG. The expression of target genes was normalized to β-actin.

### ELISA (Enzyme-Linked Immunosorbent Assay)

The concentration of IL-1β in the peritoneal lavage fluid or bone marrow macrophage culture supernatant was determined by an ELISA Kit (R&D, Minneapolis, MN, US) according to the Manufacturer's instruction.

### Neutrophil Migration Assay

Bone marrow neutrophils were isolated from Wt and α_2B_AR-Tg mice using a MojoSortTM Mouse Neutrophil Isolation Kit (Biolegend, US). The isolated α_2B_AR-Tg and Wt neutrophils were stained with CellTrace™ Violet (V440) or CellTrace™ CFSE, respectively. Labeled cells were mixed at the at the ratio of 1:1 and then adoptively transferred into MSU-pretreated (3 mg/per mouse) Wt or α_2B_AR-Tg mice. Splenocytes and PECs were harvested after 6 h. The ratio and numbers of Wt (CFSE) and α_2B_AR-Tg (V440) cells in the spleen and peritoneal fluid were determined by flow cytometry. For the *in vitro* neutrophil migration, the mixed neutrophils in Dulbecco modified Eagle medium with 10% Fetal Bovine Serum were placed in the insert of a 6-μm Transwell® plate (Thermo Scientific) at the concentration of 1 × 10^6^ cells/mL (100 μl/well). The lower chamber contained 600 ul Dulbecco modified Eagle medium with 10% Fetal Bovine Serum and MIP-2(200 ng/ml, PeproTech). After incubation for 4 h in 37°C, the cells in the insert and bottom wells were collected and the ratio of Wt (CFSE) vs. α_2B_AR-Tg (V440) cells were detected by fluorescence microscope and flow cytometry.

### Statistical Analysis

Data are presented as mean ± SEM. Group comparisons were performed using Student's *t*-test by GraphPad Prism software. *p*-values (two-tailed) below 0.05 were considered as statistically significant.

## Results

### Neutrophil Recruitment in α_2B_AR-Tg Mice Is Enhanced in Gout

**Figure 1:**
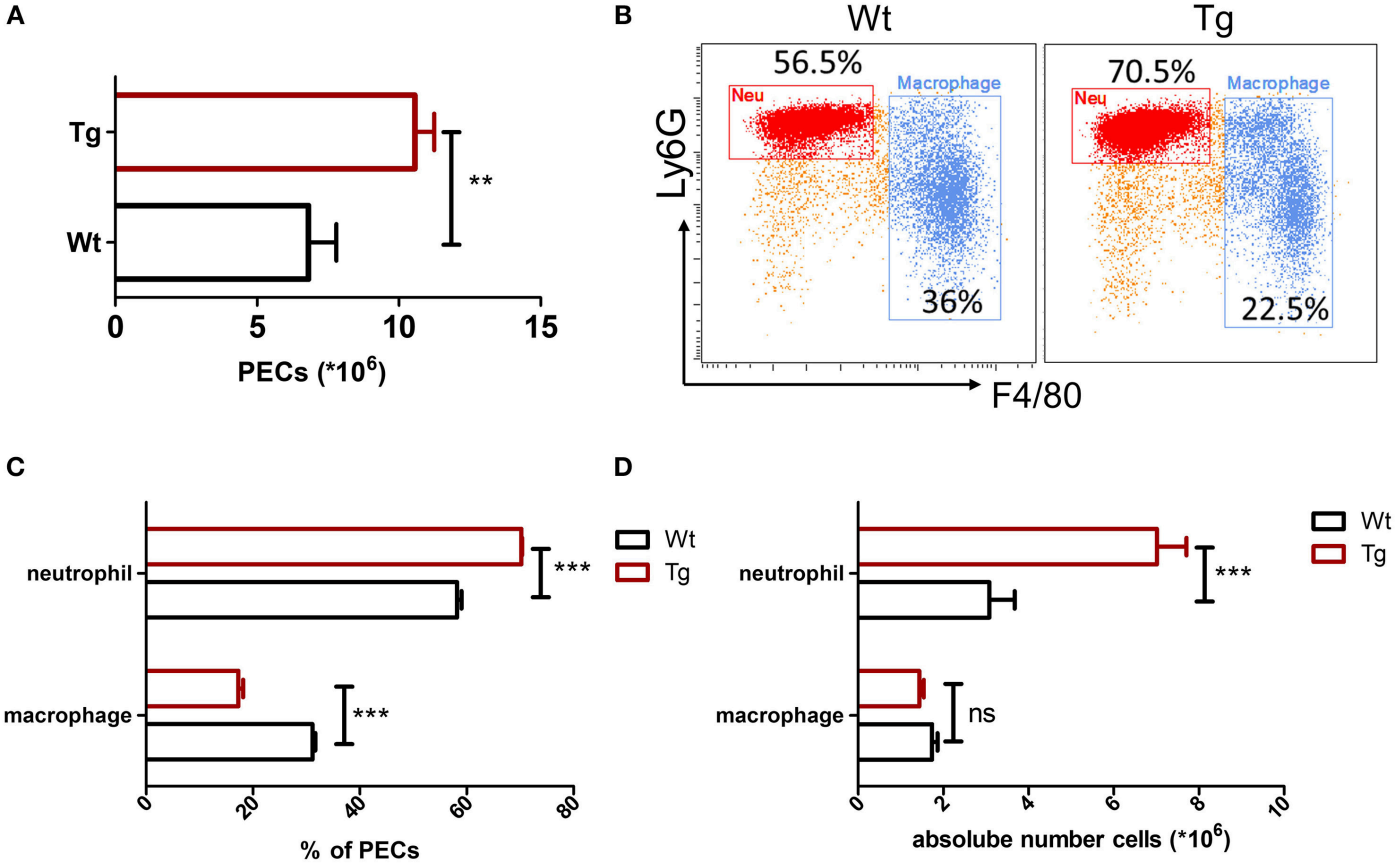
Increased neutrophil recruitment in α_2B_AR-Tg mice after MSU stimulation. Wt and α_2B_AR-Tg mice were treated with MSU to induce peritonitis. After 6 h, the total PECs were harvested. **(A)** Quantitative analysis of total number of PECs. **(B)** The PECs were stained with anti-CD11b, anti-F4/80, and anti-Ly6G antibodies. CD11b^+^ cells were gated for the analysis of macrophage (CD11b^+^Ly6G^−^F4/80^+^) and neutrophil (CD11b^+^Ly6G^+^F4/80^−^). Representative images were shown. **(C)** Statistical bar graph showing the percentages of macrophages and neutrophils in total CD11b^+^ cells. **(D)** The absolute numbers of neutrophils and macrophages in PECs. Data are represented as mean ± SEM (*n* = 6–8/group). The results shown are from one of three independent experiments. **p < 0.01, ***p < 0.001. ns, not significant.

**Figure 2:**
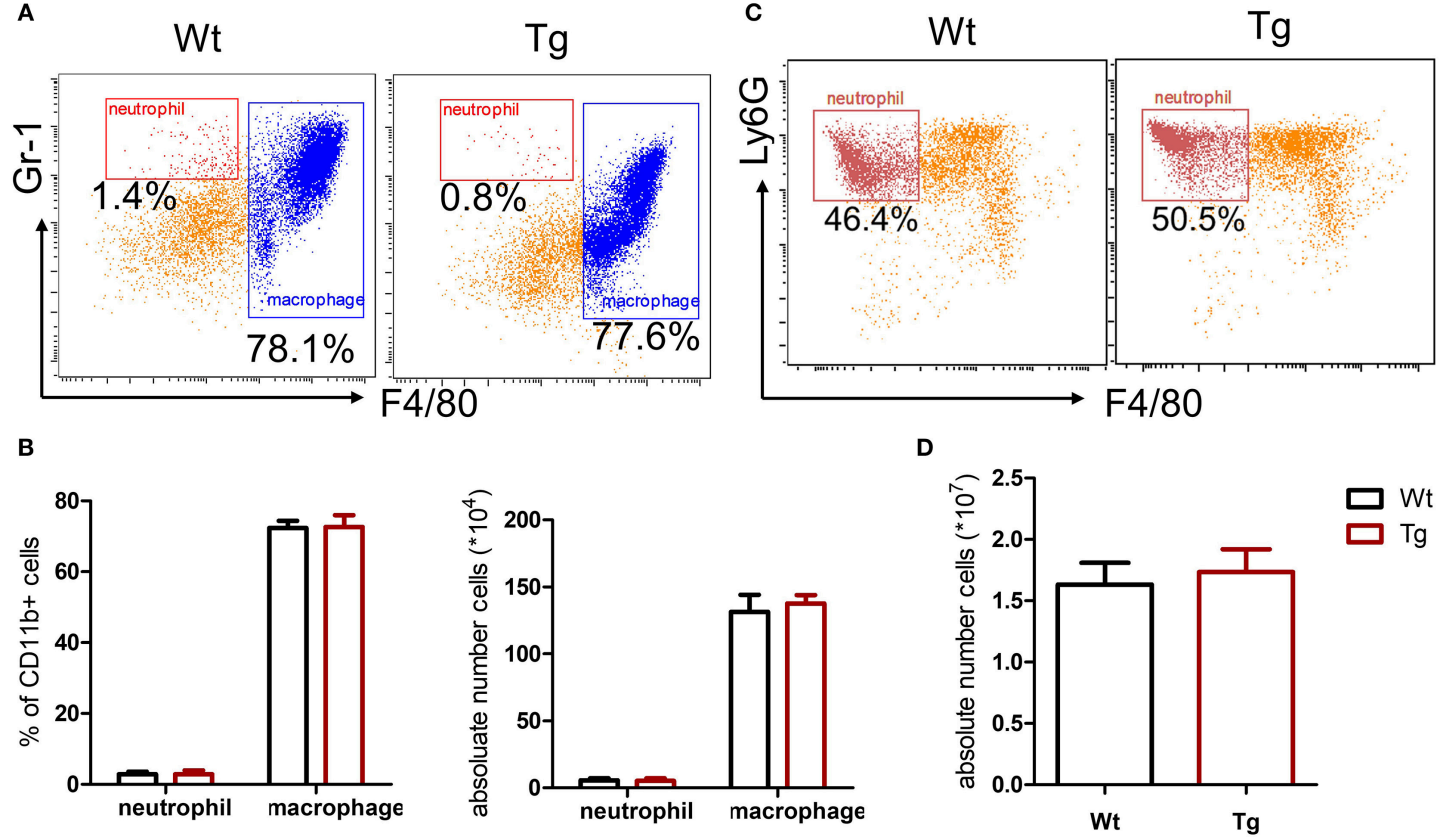
α_2B_AR over-expression does not affect the development of macrophage and neutrophil. The PECs and bone marrow cells were collected from untreated α_2B_AR-Tg and Wt mice. **(A,B)** Percentage of CD11b^+^F4/80^+^ macrophages in PECs. The cells were stained with anti-CD11b, anti-F4/80, and anti-Gr1 antibodies. The CD11b+ cells were gated for analysis. The percentage and absolute number of CD11b^+^F4/80^+^ macrophages and CD11b^+^Gr1^+^F4/80^−^ neutrophils in PECs were presented. **(C,D)** Bone marrow cells were stained with anti-CD11b, anti-F4/80, and anti-Ly6G antibodies. The percentage and absolute number of CD11b^+^Ly6G^+^F4/80^−^ neutrophils in bone marrow cells was shown. The results shown are from one of three independent experiments (*n* = 6–8/group).

Recent studies have shown a cross-communication between α_2_AR and innate immune response (3), while the role and mechanism of α_2B_AR subtype in the pathogenesis of gout are not clear. In this study, we first assessed the role of α_2B_AR in MSU-induced peritonitis, a mouse model of gout. At 6 h after MSU challenge, the total PECs were harvested for the flow cytometric analysis. Compared with the wild-type mice (Wt), α_2B_AR-Tg mice displayed a markedly elevated number of inflammatory cells in the peritoneal cavity (Figure 1A). The proportion of neutrophils, defined as CD11b^+^Ly6G^+^F4/80^−^ cells, was significantly increased in the peritoneal cavity of α_2B_AR-Tg mice. Conversely, a decreased proportion of macrophage (CD11b^+^F4/80^+^) was observed (Figures 1B,C). To further confirm the role of α_2B_AR on MSU-induced neutrophil and macrophage infiltration, the absolute number of neutrophils and macrophage in the peritoneal cavity were also calculated. The absolute number of neutrophils was considerably increased in α_2B_AR-Tg mice, while the absolute numbers of macrophages were similar in the Wt and α_2B_AR-Tg mice (Figure 1D). Furthermore, there was no differences in the frequency of macrophage and neutrophil in the peritoneal cavity (Figures 2A,B) and bone marrow (Figures 2C,D) between the α_2B_AR-Tg and Wt mice without MSU treatment. Collectively, these data demonstrated that α_2B_AR overexpression promoted the development of MSU-induced inflammation.

### α_2B_AR Does Not Affect MSU-Stimulated IL-1β Production

**Figure 3:**
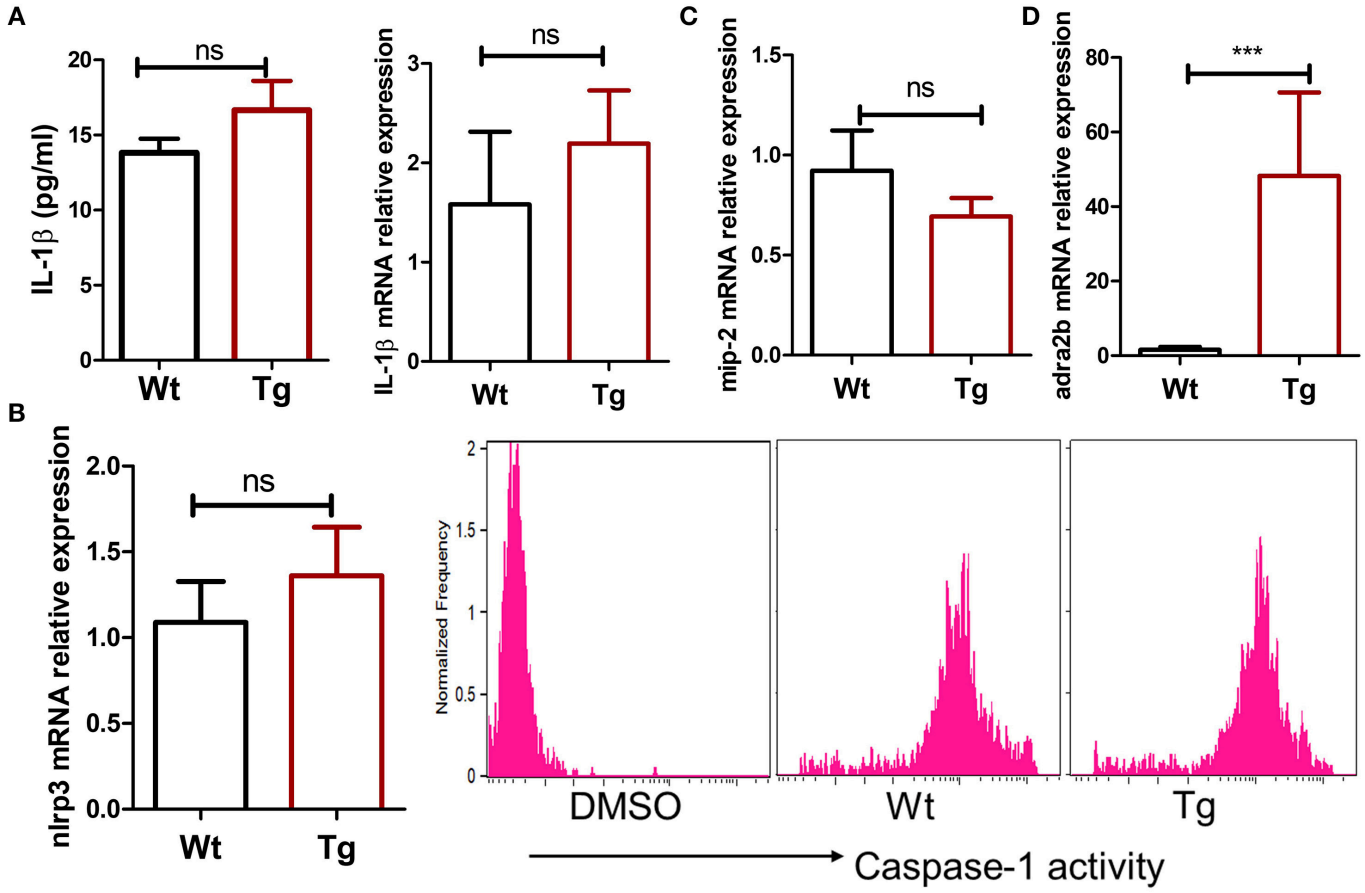
α_2B_AR overexpression does not affect IL-1β and MIP-2 production in the MSU-induced gout model. Wt and α_2B_AR-Tg mice were i.p. injected with MSU to establish the gout model. After 6 h, the peritoneal lavage fluids and PECs were harvested. **(A)** The IL-1β levels in peritoneal lavage fluid were detected by ELISA, and mRNA expression of PECs was analyzed by real-time PCR. **(B)** The caspase-1 activity in peritoneal macrophage was measured by flow cytometry analysis. DMSO negative control indicates that no caspase-1 substrates were added. **(C,D)** The mRNA levels of MIP-2 and α_2B_AR in PECs were measured by real-time PCR. The results shown are from one of three independent experiments (*n* = 6–8/group). ***p < 0.001. ns, not significant.

**Figure 4:**
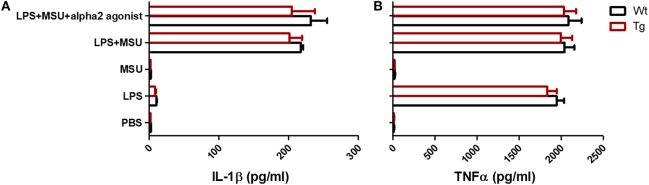
α_2B_AR overexpression has no effect on IL-1β and TNFα production by bone marrow derived macrophage. BMMs from α_2B_AR-Tg and Wt mice were treated with LPS and/or MSU, with or without the presence of alpha2 agonist (*n* = 3/group). Six hours after MSU or LPS challenge, culture supernatants were collected for the detection of IL-1β **(A)** and TNFα **(B)** concentrations. Data shown is representative of 3 independent experiments.

MSU crystals can be phagocytosed by macrophages, leading to the release of pro-inflammatory cytokines such as IL-1β, TNFα, and IL-6. Recent studies have suggested that IL-1β produced by macrophage is a key inflammatory mediator in MSU crystal-induced inflammation. Massive neutrophil infiltration induced by MSU was eliminated in IL-1 receptor knockout mice (14). Since we did not observe a change in the absolute number of macrophages in α_2B_AR-Tg mice after MSU treatment, we next examine the ability of macrophages to produce IL-1β. To explore whether the increased neutrophil recruitment in MSU-treated α_2B_AR-Tg mice is correlated with IL-1β expression, we analyzed the IL-1β levels of peritoneal lavage fluid from α_2B_AR-Tg mice and Wt mice treated with MSU. Unexpectedly, there was no significant difference in IL-1β production between α_2B_AR-Tg mice and Wt mice (Figure 3A). The maturation of active IL-1β requires inflammasome activation (19). In consistency with the result of IL-1β, α_2B_AR overexpression did not change the NLRP3 expression and caspase-1 activity (Figure 3B). Actually, a similar expression of MIP-2, the important chemokine of neutrophil, was observed in Tg and Wt mice (Figure 3C). Notably, the α_2B_AR expression of PECs was still higher in Tg mice than Wt mice after MSU challenge (Figure 3D). Next, we further investigated the effect of α_2B_AR overexpression on IL-1β production by macrophages in an *in vitro* experiment. In accordance with the *in vivo* findings, there was no difference in IL-1β expression between α_2B_AR-Tg and Wt BMMs (Figure 4A). In addition, the production of TNFα was also not affected by α_2B_AR overexpression (Figure 4B) Taken together, these results indicated that there is no significant difference in IL-1β production between α_2B_AR-Tg mice and Wt mice. The increased number of neutrophils in the peritoneal cavity of α_2B_AR-Tg mice may be a result of enhanced migration potential of neutrophils.

### α_2B_AR Overexpression Enhances Neutrophil Recruitment in a Mouse Model of Gout

**Figure 5:**
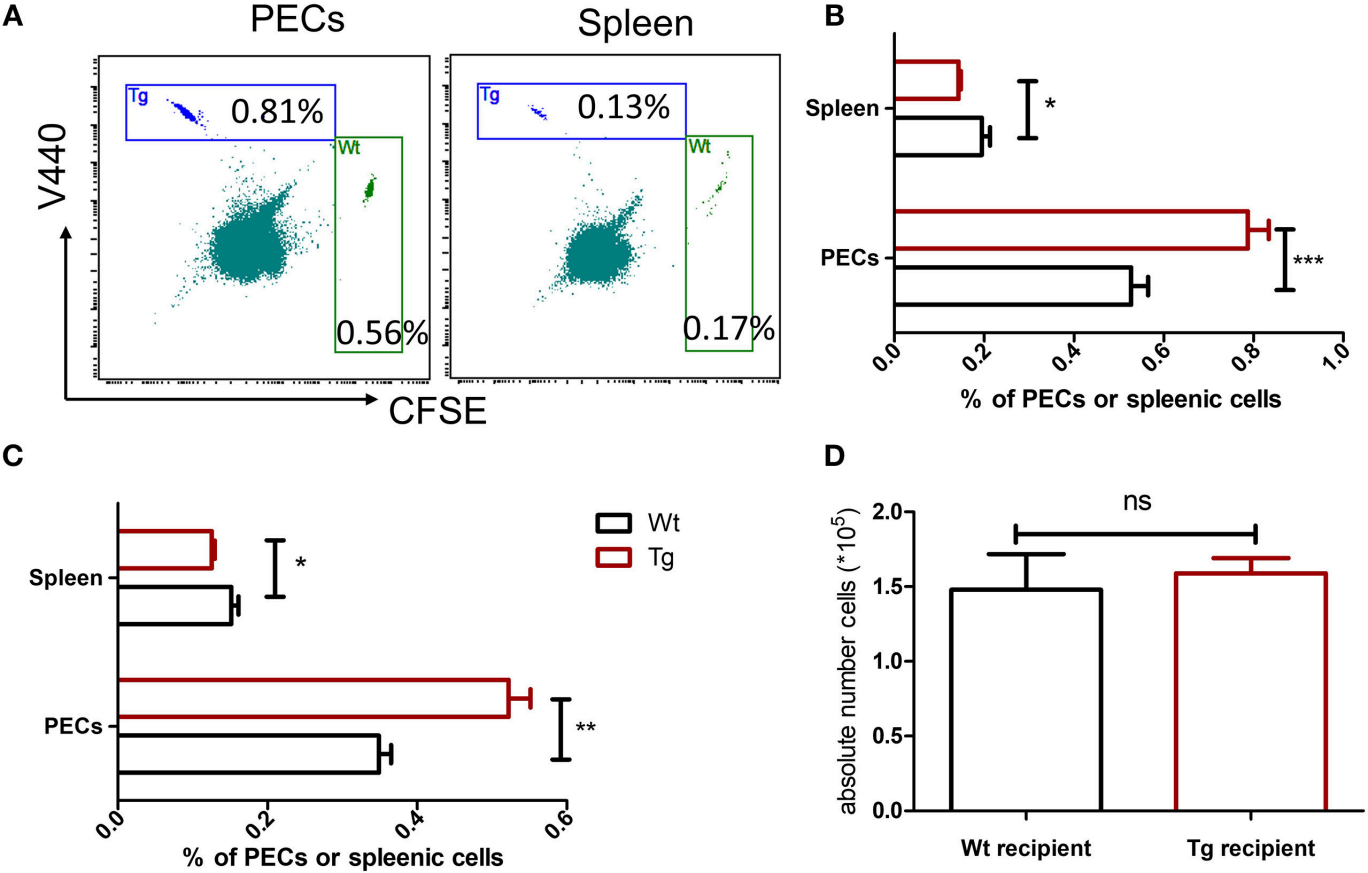
α_2B_AR enhances neutrophil recruitment in MSU-stimulated inflammation. Bone marrow neutrophils from α_2B_AR-Tg or Wt mice were stained with V440 and CFSE, respectively. Cells were then mixed at 1:1 ration, followed by i.v. injection into MSU-treated Wt mice **(A,B)** or α_2B_AR-Tg mice **(C)**. Six hours after MSU challenge, the PECs and spleen were harvested. **(A)** Representative flow cytometry analysis of neutrophil proportion in the PECs and splenic cells from MSU-treated Wt mice. **(B,C)** Percentages of neutrophils in PECs and spleen from MSU-treated Wt **(B)** and α2BAR-Tg mice **(C)** were analyzed. **(D)** The peritoneal fluid was collected from Wt and α2BAR-Tg recipients and the absolute number of adoptively transferred neutrophils were analyzed. Data shown are representative of 3 independent experiments (*n* = 5/group). *p < 0.05, **p < 0.01, ***p < 0.001. ns, not significant.

To test whether the expression of α_2B_AR on neutrophils regulates their migration, neutrophils isolated from α_2B_AR-Tg mice and Wt mice were labeled with different fluorescent dyes, respectively, followed by adoptive transfer into MSU-treated recipients. Equal numbers of V440-labeled α_2B_AR-Tg neutrophils and CFSE-labeled Wt neutrophils were mixed and transferred into MSU-pretreated Wt mice. The PECs and splenocyes were harvested for the flow cytometric detection of V440-labeled α_2B_AR-Tg and CFSE-labeled Wt neutrophils (Figure 5A). As expected, there were more α_2B_AR-Tg neutrophils in the peritoneal cavity of the Wt recipients (Figure 5B). Moreover, α_2B_AR-Tg neutrophils had an enhanced migration into the peritoneal cavity of MSU-treated α_2B_AR-Tg recipients (Figure 5C). To further examine if the expression of α_2B_AR in other tissues or cell types would affect neutrophil recruitment, we compared the migration of neutrophils into the peritoneal cavity of Wt vs. α_2B_AR-Tg mice. We found the absolute number of neutrophils migrated to the peritoneal cavity of α_2B_AR-Tg recipients was similar to that migrated into the peritoneal cavity of Wt recipients (Figure 5D), suggesting the overexpression of α_2B_AR in other tissue or cell types did not affect the migratory ability of neutrophils. These data indicated that α_2B_AR overexpression on neutrophils promotes its migratory ability.

### α_2B_AR Overexpression Enhances the Migratory Capacity of Neutrophil *in vitro*

**Figure 6:**
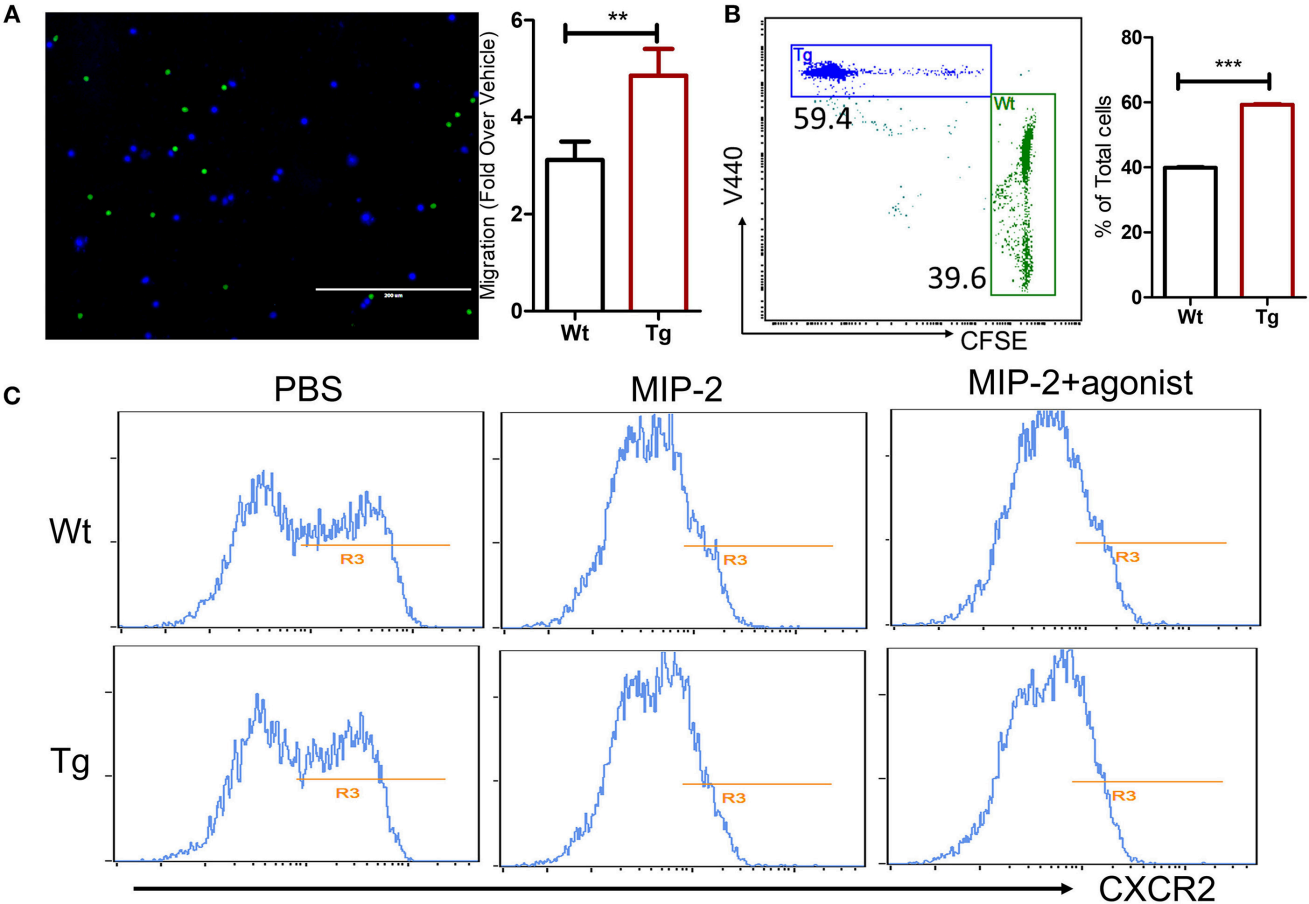
α_2B_AR overexpression enhances *in vitro* neutrophils migration. Neutrophils were isolated from α2BAR-Tg and Wt bone marrows and stained with V440 (blue) and CFSE (green), respectively. Labeled cells were mixed at 1:1 ration and used for the Transwell® migration assay in response to MIP2. **(A)** Upper panel, Representative fluorescent microscopic image showing α2BAR-Tg (Blue) and Wt (Green) neutrophils migrating through the membrane. Lower panel, quantitative analysis showing the migration of α2BAR-Tg and Wt neutrophils. **(B)** Representative dot plot (upper panel) and quantitative analysis (lower panel) showing the percentages of V440-labeled cells (α2BAR-Tg) and CFSE-labeled cells (Wt) in the bottom well of the Transwell® plate. **(C)** The Cxcr2 expressions on bone marrows neutrophil with or without treatment of MIP-2 or MIP-2 plus α2BAR agonist clonidine hydrochloride were analyzed by flow cytometry. Data shown are representative of 3 independent experiments. **p < 0.01, ***p < 0.001.

Because lots of factors can affect the neutrophil migration during the development of inflammation *in vivo*, the migratory capacity of α_2B_AR-Tg neutrophils was further investigated *in vitro* to exclude the microenvironmental factors. CXCR2 has a central role in the recruitment of neutrophils. We next examined the migration of the neutrophils toward CXCR2 ligand MIP-2. In consistency with the *in vivo* result, α_2B_AR overexpression on neutrophils increased their chemotaxis toward MIP-2 in the *in vitro* Transwell® assay (Figures 6A,B). We next examined the expression level of CXCR2 on neutrophils. No significant difference in CXCR2 expressions on bone marrow neutrophils was observed between α_2B_AR-Tg and Wt mice (Figure 6C). It has been shown that the binding with MIP-2 induces CXCR2 internalization. In consistency, we here also observed a decreased expression on neutrophil surface after MIP-2 treatment in both Wt and Tg mice. There was also no significant difference in cell surface expression of CXCR2 after MIP-2 stimulation, suggesting that α_2B_AR does not affect CXCR2 internalization (Figure 6C). In addition, α_2B_AR activation by agonist clonidine hydrochloride did not affect the expression of CXCR2, further suggesting that the enhanced neutrophil migration was independent of CXCR2 expression (Figure 6C). These data strongly suggested that the increased migratory ability of α_2B_AR-Tg neutrophils was independent of CXCR2.

## Discussion

In this study, we showed that MSU-induced inflammation was enhanced in α_2B_AR-Tg mice, characterized by increased neutrophil infiltration. α_2B_AR overexpression did not affect the IL-1β production by macrophage, while the migratory ability of neutrophils was significantly enhanced in α_2B_AR-overexpressing neutrophils.

Increasing evidence show that adrenergic receptors have a close relationship with immune system. All the three families of AR have already been reported to play a role in regulating innate immune responses and immune-related diseases (2, 20). For instance, βARs are highly and widely expressed in immune cells. They regulate a number of functions, including lymphocytes homing and maturation (8, 21, 22). Previous studies have also shown a negative influence of norepinephrine on NK cell activity via β_2_ARs (23). In our study, we found for the first time that there was a significantly increased number of inflammatory cells in peritoneal cavity of α_2B_AR-Tg mice upon MSU treatment. IL-1β is considered as a critical pro-inflammatory cytokine in the gout, with a wide range of systemic and local effects. In humans, the production of IL-1β by resident macrophages is stimulated by MSU crystal and plays as a crucial role in the pathogenesis of gout (14). Moreover, IL-1β has the ability to induce neutrophilia and neutrophil migration (24). It has been reported that exposure of macrophages to lipopolysaccharide led to a release of catecholamines and there is an autocrine/paracrine self-regulatory mechanism in catecholamine production by macrophages during inflammation (25). Blockade of α_2_AR or catecholamine-generating enzymes greatly suppressed LPS-induced inflammation (25). Catecholamines has been shown to be able to promote NF-κB signaling in macrophages via activating α_2_AR, leading to an amplification of the acute inflammatory response (26). However, we did not observe an increased IL-1β production and caspase-1 activity in the α_2B_AR-overexpressing macrophages in our study. These data demonstrated that increased cytokines production induced by catecholamines may be independent of α_2B_-AR stimulation.

As an early event in immune response against invading pathogens, accumulation of neutrophils was also an early and critical step in sterile inflammation, such as gout and ischemia/reperfusion (27, 28). Inflammatory stimuli induce neutrophil mobilization from bone marrow, resulting in neutrophilia and neutrophil infiltration into the affected local area (29). In consistency with this, the number of neutrophil is significantly increased in the patients with acute gout. Both vagal parasympathetic and sympathetic signals have been shown to regulate inflammation through activating cholinergic receptors on macrophage and neutrophils (3, 30–32). It has been demonstrated that norepinephrine can induce immediate neutrophil mobilization through acting on α-AR (33), and leukocyte-expressing β_2_-AR has been found to be essential for survival after acute myocardial injury (34). Propranolol, a β_1_-AR/β_2_-AR inhibitor, is able to reduce the numbers of circulating neutrophils, suppress neutrophil infiltration into colonic tissues, and attenuate the colonic tissue damage in IBD (35). Here, the increase of the inflammatory cell number in MSU-treated α_2B_AR-Tg mice was identified to a result of enhanced migratory ability of neutrophils as the absolute number of macrophage did not change. Furthermore, the number of neutrophils in bone marrow was similar between treatment naive α_2B_AR-Tg and WT mice, excluding the possibility that α_2B_AR may regulate neutrophil differentiation. The regulation of neutrophil function by SNS may vary in different diseases. Our findings indicate that α_2B_AR overexpression might play a critical role in the development of gout through promoting neutrophil recruitment into the inflammatory sites.

The migration process of neutrophil includes the following steps. The initial step of neutrophils is movement from the central stream to the periphery of a vessel, which allows the cell surface molecular interaction between neutrophil and vessel endothelial cell and then neutrophil rolling on the endothelial cells. After that, a chemoattractant gradient produced by local inflammation elicits the transmigration process of neutrophils (27). In our study, no difference in IL-1β production was observed between Wt and α_2B_AR-Tg mice. In addition, mixed Wt and α_2B_AR-Tg neutrophils displayed different migratory ability in the same environment, regardless in Wt or α_2B_AR-Tg recipients. Furthermore, Wt and α_2B_AR-Tg neutrophils had an enhanced migration toward MIP2, while the CXCR2 receptor expression was similar to Wt neutrophils. These data suggest that the enhanced migratory ability in α_2B_AR-overexpressing neutrophils is independent of chemokine and chemokine receptors. Further investigations are required to understand the exact mechanisms by which α_2B_AR signals regulate the mobility of neutrophils. Taken together, we demonstrated that α_2B_AR is involve in the progress of MSU-induced inflammation and α_2B_AR might be a promising therapeutic target for gout.

## Data Availability

All datasets generated for this study are included in the manuscript and/or the supplementary files.

## Author Contributions

LD, XR, and JZ conceived the project, designed and carried out some experiments, analyzed all data, and wrote the paper. JC were responsible for design and performance of experiments, and contributed to analyzing data and writing the paper. YW, MR, and YT helped in performing all the experiments, analyzing all data, generating figures of the data. All the authors read, critically revised, and agreed to be accountable for the content of manuscript.

### Conflict of Interest Statement

The authors declare that the research was conducted in the absence of any commercial or financial relationships that could be construed as a potential conflict of interest.
